# Advances in platinum-based drug development for cervical cancer therapy

**DOI:** 10.3389/fphar.2026.1794356

**Published:** 2026-10-28

**Authors:** Donghan Shao, Ping Gao, Qian Wang, Yuqin Ma, Irina Kolesnik, Vladimir Potkin, Leijiao Li, Yongjun Teng

**Affiliations:** 1 School of Chemistry and Environmental Engineering, Changchun University of Science and Technology, Changchun, China; 2 Institute of Physical Organic Chemistry, National Academy of Sciences of Belarus, Minsk, Belarus; 3 Department of Obstetrics and Gynecology, Wenling Maternal and Child Healthcare Hospital, Taizhou, China

**Keywords:** antitumor efficacy improvement, cervical cancer, drug-controlled release, nano-delivery system, platinum-based drug

## Abstract

Cervical cancer remains a predominant malignancy of the female reproductive system and a critical global health challenge. Traditional platinum-based agents such as cisplatin, carboplatin, and oxaliplatin, are cornerstones in postoperative adjuvant therapy, management of locally advanced cases, and systemic treatment of recurrent/metastatic disease. However, their clinical efficacy is frequently hampered by severe systemic toxicities and the emergence of chemoresistance. To address these bottlenecks, diverse nano-delivery platforms have been engineered to enhance bioavailability and mitigate adverse effects. This review systematically delineates the evolution of first-to third-generation platinum drugs, elucidating their molecular mechanisms of action specifically within the landscape of cervical cancer. Furthermore, we critically evaluate the clinical application of these agents and provide a comprehensive synthesis of recent advances in nano-delivery systems, including targeted and stimuli-responsive carriers. By bridging traditional pharmacology with cutting-edge nanotechnology, this review offers strategic insights into achieving precise delivery and controlled release, potentially defining new paradigms for the next-generation of cervical cancer therapeutics.

## 1 Introduction

Cervical cancer is one of the common malignancies that pose a significant threat to women’s life and health, with a particularly high incidence among women aged 35-40 (Francoeur et al., 2025). In recent years, cervical cancer screening and human papillomavirus (HPV) vaccination have alleviated the incidence of cervical cancer to a certain extent, but its incidence rate still ranks first among malignancies of the female reproductive system, and exhibits a trend toward youthfulness. According to the latest GLOBOCAN 2022 estimates released in 2024, there were approximately 661044 new cases and 348186 deaths globally (Bray et al., 2024). Although it ranks fourth among the most common cancers in women worldwide, cervical cancer remains the most prevalent malignant tumor of the female reproductive system in many developing countries, and nearly 90% of deaths worldwide occur in low- and middle-income countries. Currently, clinical interventions for cervical cancer include surgical resection, radiation therapy, and chemotherapy, often supplemented by targeted therapy and immunotherapy (Ye et al., 2023). Among them, platinum-based chemotherapy regimens have always played an irreplaceable role in preventing postoperative recurrence, controlling locally advanced tumors, and providing systemic treatment for recurrent or metastatic lesions.

Currently, the standard of systemic treatment for cervical cancer is highly dependent on tumor stage and pathological characteristics. For locally advanced cervical cancer (LACC), cisplatin-based concurrent chemoradiotherapy (CCRT) remains the “gold standard” in clinical treatment. However, in the first-line treatment of recurrent or metastatic disease, clinical practice has shifted towards more complex combination regimens, including the introduction of anti-angiogenic drugs (such as bevacizumab) or immune checkpoint inhibitors (such as pembrolizumab) (Liang et al., 2024; Jiang et al., 2025).

Based on their chemical structure and clinical applications, these drugs can be divided into three generations. Cisplatin remains the gold standard, while subsequent drugs such as carboplatin and oxaliplatin were developed to overcome their systemic toxicity and drug resistance issues (Dasari et al., 2022; de Vries et al., 2020; Song et al., 2022; Rades et al., 2023; Negi et al., 2020; Shahsavani et al., 2025; Dehghani et al., 2025). In addition to cisplatin, carboplatin, and oxaliplatin, which are widely used globally, derivatives such as nedaplatin and lobaplatin are also being integrated into clinical practice in specific regions, such as East Asia, due to their unique toxicity characteristics. For example, carboplatin and oxaliplatin are often used in combination chemotherapy for relapsed or metastatic diseases to reduce systemic side effects in patients.

Advances in nanotechnology and molecular pharmacology, nano-delivery systems can enhance the tumor specificity of drugs via both passive and active targeting approaches, while simultaneously improving the bioavailability, stability and targeting ability of therapeutics (Pan et al., 2022). The main strategies for conjugating platinum drug with delivery carriers include physical encapsulation, chemical covalent bonding, and electrostatic interaction. At present, platinum-based drugs can be conjugated with inorganic materials (e.g., carbon materials, mesoporous silica nanoparticles and gold nanomaterials) and organic materials (e.g., polymers, dendrimers, and liposomal micelles) via physical encapsulation, chemical covalent bonding, or electrostatic interaction to achieve drug delivery (Kapadne et al., 2024; Kalayil et al., 2024; Stępień et al., 2025; Santos et al., 2024) Despite the promise of nanotechnology, designing ideal delivery platforms for cervical cancer requires overcoming hurdles such as optimizing drug loading capacity, minimizing premature leakage in systemic circulation, and ensuring spatiotemporally controlled release at the tumor site. Nevertheless, the design of platinum-based drug delivery systems still faces the following challenges: high-efficiency drug loading capacity, reliable premature leakage prevention performance, precise lesion-targeting capability, and controllable drug release efficiency.

This review provides a comprehensive overview on the development process, the anti-tumor mechanisms, and the mechanisms of drug resistance of platinum-based drugs. It also elaborates on the current clinical application status and optimization strategies of platinum-based metal drugs in the treatment of cervical cancer, aiming to provide references and insights for future translational medicine research on platinum-based drugs.

## 2 Development process and antitumor mechanism

Given the significant role of platinum-based drugs in the treatment of cervical cancer, we summarize the evolutionary process of these drugs from accidental discovery to systematic development and present their anti-tumor mechanisms (Figure 1).

**FIGURE 1:**
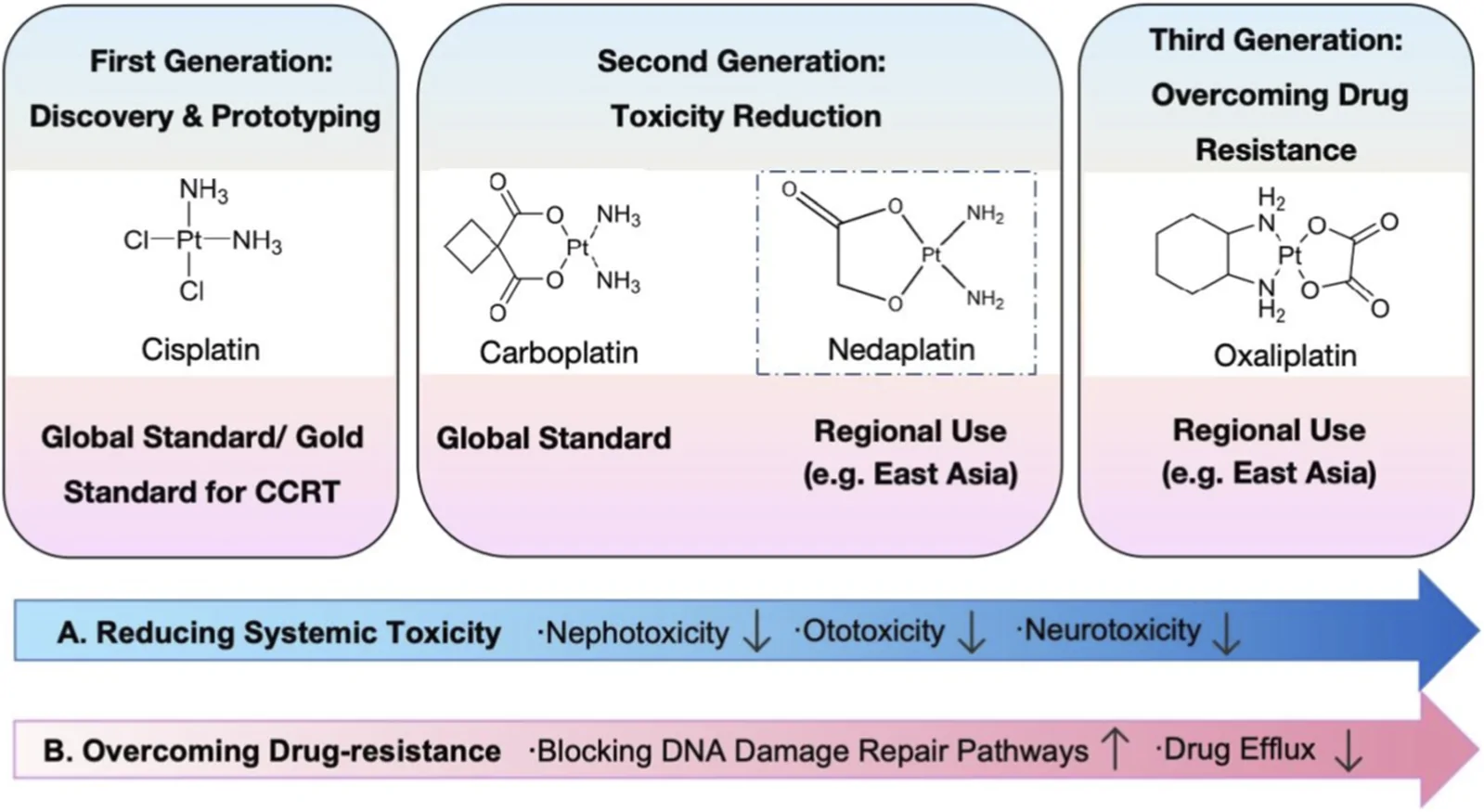
The structural evolution and clinical categorization of major platinum-based agents.

### 2.1 Cisplatin: discovery and the gold standard for CCRT

Cisplatin (cis-[PtCl_2_(NH_3_)_2_]) stands as the pioneering heavy metal coordination compound with broad-spectrum antitumor activity. Since its FDA approval in 1978, it has remained the global gold standard for concurrent chemoradiotherapy (CCRT) in cervical cancer (Peyrone, 1844; Rosenberg et al., 1969; Mitsunaga et al., 2011).

The cytotoxic mechanism of cisplatin involves the formation of DNA cross-links that inhibit transcription and replication. Upon cellular entry via copper transporters (CTR1), the low intracellular chloride environment triggers the substitution of chloride ligands with water molecules, forming electrophilic aqua-complexes (Holzer et al., 2006) (Figure 2). These positively charged species are subsequently attracted to the negatively charged nuclear DNA and interact with the nucleophilic centers of DNA purine bases (specifically the N7 sites of guanine or adenine). The resulting platinum-DNA adducts bend and distort the DNA structure, inhibit transcription, trigger cell apoptosis, and ultimately lead to cell death (Rocha et al., 2018).

**FIGURE 2:**
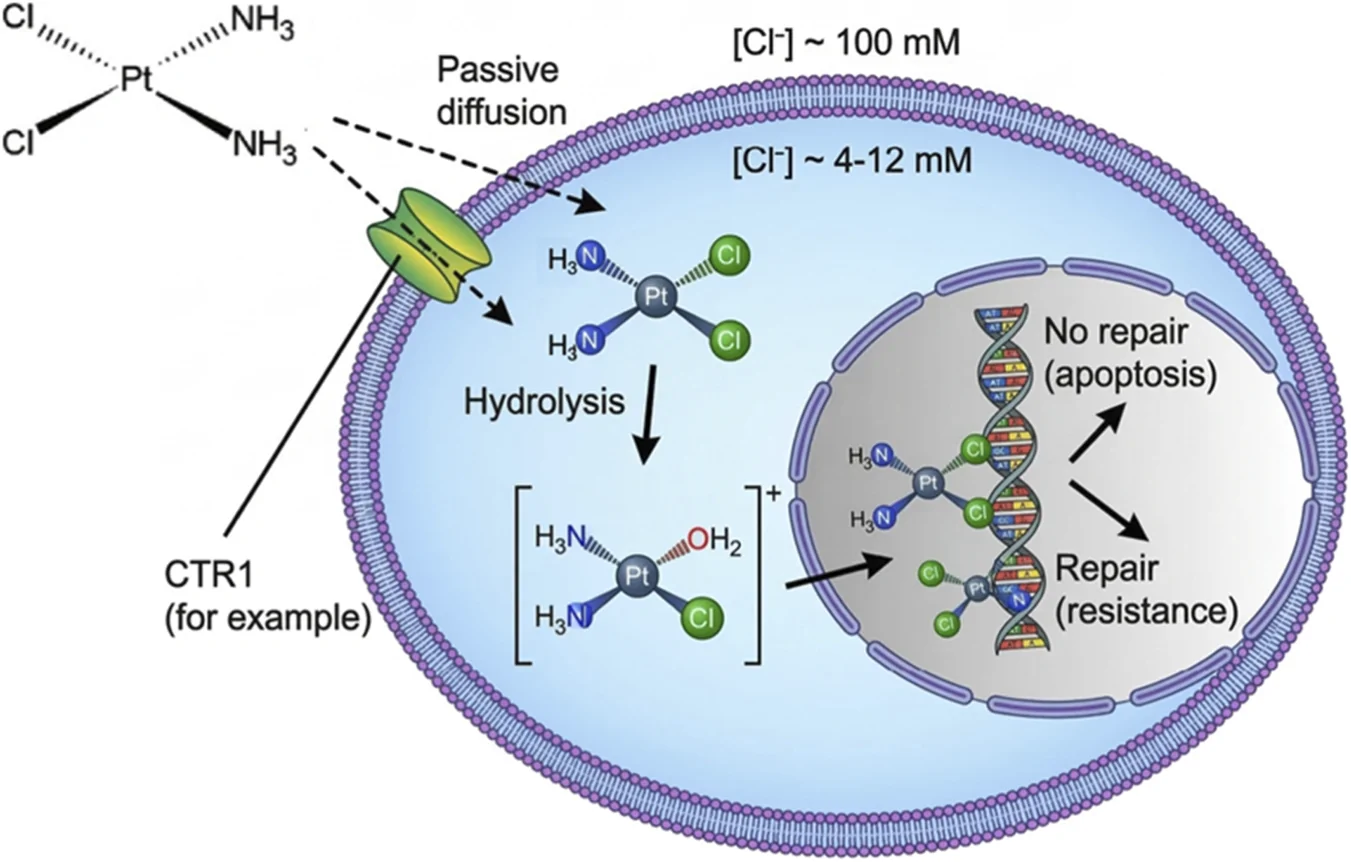
The structure and mechanisms of action of cisplatin (Browning et al., 2017).

Despite its clinical efficacy, cisplatin is limited by significant systemic toxicities, most notably nephrotoxicity, which affects approximately 30% of patients at standard therapeutic doses (50–75 mg/m^2^). Additional complications include neurotoxicity, ototoxicity, and myelosuppression, alongside the persistent challenge of intrinsic and acquired resistance (Chen and Chang, 2019).

### 2.2 Structural evolution for toxicity reduction: carboplatin and nedaplatin

To mitigate these adverse effects, second-generation analogues with modified leaving groups were developed to enhance patient tolerability. Carboplatin, characterized by the replacement of chloride ligands with a cyclobutene-1,1-dicarboxylate moiety, represents a significant advancement in structural optimization. This modification increases water solubility 16-fold and slows the drug’s activation kinetics, resulting in a substantially reduced incidence of nephrotoxicity, neurotoxicity, and emesis compared to cisplatin (Calvert et al., 1982). While carboplatin is recognized as a global standard for patients with renal impairment or cisplatin intolerance, its clinical utility is often constrained by dose-limiting myelosuppression and cross-resistance with cisplatin, as both agents share the same diammine carrier group. Similarly, Nedaplatin features a glycolate leaving group that enhances solubility and reduces renal toxicity. Although integrated into clinical practice primarily in East Asia as a regional alternative for advanced or recurrent cases, nedaplatin exhibits a similar mechanism of DNA adduct formation and remains effective in some cisplatin-resistant phenotypes, though it has not reached universal standard status.

### 2.3 Structural evolution to overcome resistance: oxaliplatin and lobaplatin

The third evolutionary phase focused on bypassing the DNA repair mechanisms responsible for cisplatin resistance. Oxaliplatin incorporates a 1,2-diaminocyclohexane (DACH) ligand, which forms bulkier, more rigid DNA adducts compared to earlier generations. These unique adducts are less easily recognized and repaired by the nucleotide excision repair (NER) system or mismatch repair (MMR) proteins, allowing oxaliplatin to maintain activity in cisplatin-resistant cell lines (Wheate et al., 2010; Quintão et al., 2019). Since its FDA approval in 2002, oxaliplatin has become a cornerstone in combination regimens for recurrent cervical cancer. Parallel to this, Lobaplatin-another DACH-containing derivative approved in China-offers an alternative for patients with established resistance or severe gastrointestinal intolerance to standard therapies. By substituting the leaving groups and incorporating bulky ligands, these third-generation agents provide a strategic approach to extending the therapeutic window and overcoming the molecular barriers of traditional platinum therapy.

## 3 Mechanisms of platinum resistance in cervical cancer

To optimize platinum-based therapy, a comprehensive understanding of the multifaceted resistance mechanisms is imperative. These mechanisms are not isolated but rather form an interconnected network that diminishes the therapeutic efficacy of platinum agents in cervical cancer through the following dimensions.

### 3.1 Impaired cellular accumulation and increased efflux

The therapeutic threshold of cisplatin is primarily determined by its net intracellular concentration. Reduced expression of the copper transporter CTR1, the primary influx protein for cisplatin, significantly limits drug entry into cervical cancer cells (Xiao et al., 2024). Concurrently, the ATP-binding cassette (ABC) transporter family, particularly P-glycoprotein (MDR1/P-gp) and the copper-transporting ATPases (ATP7A and ATP7B), are frequently overexpressed in resistant cervical tissues. These pumps actively sequester platinum into secretory vesicles or export it from the cell, effectively lowering the cytosolic drug payload before it can reach its nuclear target (Zhao et al., 2025).

### 3.2 Enhanced intracellular detoxification

Once inside the cytoplasm, platinum ions are vulnerable to sequestration by nucleophilic scavengers. In resistant cervical cancer cells, the upregulation of the glutathione (GSH) system and metallothioneins (MTs) acts as a potent detoxification shield. GSH, often mediated by high levels of glutathione S-transferase (GST), forms stable platinum-thiol conjugates that are chemically inactive and easily excrete (Bhattacharjee et al., 2022). This pre-nuclear neutralization prevents the formation of lethal DNA adducts, representing a major hurdle for first-line cisplatin efficacy (Wang et al., 2024).

### 3.3 Augmentation of DNA repair pathways

The hallmark of platinum cytotoxicity is the formation of intra-strand and inter-strand DNA cross-links. However, resistant cells counteract this damage by mobilizing robust DNA repair machinery. Nucleotide Excision Repair (NER), specifically through the upregulation of ERCC1, is a primary driver of resistance in cervical cancer, as it efficiently identifies and excises bulky platinum-DNA adducts (Doll et al., 2010). Furthermore, alterations in the DNA mismatch repair (MMR) system, often characterized by the loss of MLH1 or MSH2—allow cells to tolerate genomic lesions that would otherwise trigger cell cycle arrest, leading to a “damage tolerance” phenotype (Xu et al., 2025).

### 3.4 Evasion of programmed cell death

The ultimate success of platinum therapy relies on the induction of apoptosis. Resistance frequently arises from mutations in the TP53 gene or dysregulation of its downstream signaling, which impairs the cell’s ability to initiate apoptosis in response to genomic stress (Zhou et al., 2025). Additionally, the overexpression of anti-apoptotic proteins such as Bcl-2 and XIAP, coupled with the downregulation of pro-apoptotic factors like Bax, shifts the cellular balance toward survival, enabling cervical cancer cells to persist despite extensive platinum-induced damage (Han et al., 2017).

### 3.5 Influence of the tumor microenvironment (TME)

The unique physiological landscape of cervical tumors further complicates drug delivery and response. Hypoxia, a common feature of locally advanced cervical cancer, induces the expression of hypoxia-inducible factor 1-alpha (HIF-1α), which promotes the epithelial-mesenchymal transition (EMT) and stemness, both of which are strongly correlated with multidrug resistance (Chen et al., 2024). Moreover, the acidic and poorly vascularized TME limits the physical penetration of platinum agents and alters their chemical activation, creating a sanctuary for resistant tumor sub-population (He et al., 2025).

## 4 Application of platinum-based drugs in the treatment of cervical cancer

Based on the evolution of the pharmacological mechanisms and structures of the third-generation platinum-based drugs (including the first-generation cisplatin; the second-generation carboplatin and nedaplatin; and the third-generation oxaliplatin and lobaplatin), they have gradually formed differentiated application scenarios and therapeutic characteristics in the clinical treatment of cervical cancer. In this section, we will systematically summarize the specific performance and application strategies of platinum-based drugs across generations in cervical cancer therapy by integrating existing clinical research findings (Table 1).

**TABLE 1:** Summary and comparison.

Platinum drugs	Generation	Clinical application	Representative program	Efficacy	Key benefits	Toxicities and limitations
Cisplatin	first generation	Concurrent chemoradiotherapy (CCRT) for locally advanced cervical cancer elderly patients (Whitney et al., 1999)	cisplatin + radiotherapy (RT) postoperative adjuvant therapy	significantly improves survival rates	exact efficacy and sufficient clinical evidence (Rose et al., 2007)	Severe nephrotoxicity, neurotoxicity, ototoxicity, and gastrointestinal reactions primary and secondary drug resistance (Xiang and Kidd, 2019)
Carboplatin	second generation	Patients who cannot tolerate cisplatin (e.g., frail, elderly, or those with comorbidities) Patients with renal insufficiency	Weekly carboplatin + pelvic external beam radiotherapy (Sebastião et al., 2016)	Non-inferior to cisplatin	Better tolerability (lower non-hematologic toxicity)	Myelosuppression (more pronounced than cisplatin) (Mori et al., 2010) cross-resistance with cisplatin
Nedaplatin	second generation	Concurrent chemoradiotherapy for locally advanced cervical cancer High-risk patients with para-aortic lymph node metastasis	Nedaplatin + concurrent radiotherapy	Survival is comparable to cisplatin, with a trend toward longer progression-free survival	Gastrointestinal reactions and nephrotoxicity are significantly lower than cisplatin; highly water-soluble (Yang et al., 2022); no pre-hydration is required	The toxicity spectrum is similar to that of cisplatin, but to a lesser degree; bone marrow suppression is still a common adverse reaction (Liu et al., 2016)
Oxaliplatin	third generation	Cisplatin-resistant recurrent or metastatic cervical cancer	Oxaliplatin + paclitaxel	Effective in previously treated/potentially resistant patients	The drug resistance spectrum is different from that of cisplatin, and it can overcome cisplatin resistance; it has a synergistic effect with paclitaxel (Kuo et al., 2010)	Neurotoxicity is the main dose-limiting toxicity; efficacy remains limited as a second-line treatment
Lobaplatin	third generation	Cisplatin-intolerant or resistant locally advanced cervical cancer	Paclitaxel + lobaplatin	The efficacy is equivalent to that of cisplatin regimen and is replaceable (Li et al., 2014)	Significantly lower gastrointestinal toxicity than cisplatin; incomplete cross-resistance with cisplatin	Higher hematologic toxicity (Tian et al., 2021) (such as bone marrow suppression)

### 4.1 Application of cisplatin

As a classic first-line chemotherapeutic agent for cervical cancer, cisplatin exerts potent antitumor effects by inducing DNA cross-linking in tumor cells. Cisplatin has proven effective in postoperative adjuvant therapy and locally advanced concurrent chemoradiotherapy, and is an indispensable core drug in the clinical treatment system.

Whitney et al. compared the therapeutic efficacy of different regimens in 368 patients with stage IIB-IVA cervical cancer (with negative para-aortic lymph nodes). The results demonstrated that a regimen of whole-pelvis radiotherapy combined with cisplatin and 5-fluorouracil significantly improved overall survival (OS) and progression-free survival (PFS) compared to a regimen combined with hydroxyurea. Specifically, the hazard ratio (HR) for OS was 0.74 (95% CI 0.58–0.95, P = 0.018), and the HR for PFS was 0.79 (90% CI 0.62–0.99, P = 0.033). Furthermore, the cisplatin-based group exhibited a significantly lower incidence of severe leukopenia compared to the hydroxyurea group (4% vs. 24%) (Whitney et al., 1999). This clinical evidence supports the core grouping framework of our present study, which evaluates outcomes between a cisplatin group and a non-cisplatin group. Rose et al. conducted a clinical trial involving 526 patients with stage IIB-IVA cervical cancer and no para-aortic lymph node metastasis. Patients were randomized to receive whole pelvic radiotherapy with three concurrent chemotherapy regimens: cisplatin monotherapy (Group 1), cisplatin plus 5-fluorouracil and hydroxyurea (Group 2), or hydroxyurea monotherapy (Group 3). Compared with Group 3, the relative risks of death were 0.57 (95% CI: 0.42–0.78) for Group 1 and 0.55 (95% CI: 0.40–0.75) for Group 2. Similarly, the relative risks of disease progression were significantly lower in the cisplatin-containing arms, at 0.61 (95% CI: 0.44–0.85) for Group 1 and 0.58 (95% CI: 0.41–0.81) for Group 2, relative to the hydroxyurea-only group (Rose et al., 2007). According to a SEER-Medicare database-based study by Xiang et al., the proportion of those receiving cisplatin treatment is low, but the 5-year cervical cancer-specific mortality rate of patients receiving cisplatin combined with radiotherapy is significantly lower than that of those who did not use cisplatin. This finding provides critical evidence to support cisplatin-based concurrent chemoradiotherapy as the standard treatment regimen for this patient population (Xiang and Kidd, 2019).

### 4.2 Application of carboplatin and nedaplatin

As for patients with cisplatin intolerance, carboplatin and other second-generation platinum compounds can be used as key alternative therapeutic strategies. They can enhance treatment safety and improve tolerance, thereby expanding the patient population that can benefit from the treatment.

Ana Morais Sebastiao et al. compared the efficacy of cisplatin and carboplatin combined with concurrent external pelvic radiotherapy in the treatment of stage IIB-IVA cervical cancer (Sebastião et al., 2016). No statistically significant differences were observed between the two groups in terms of 3-year overall survival, progression-free survival, and overall response rate, indicating that carboplatin is comparable to cisplatin in therapeutic efficacy. Furthermore, elderly patients with multiple comorbidities in the carboplatin group exhibited favorable treatment tolerability, with no treatment delays or interruptions attributed to toxic reactions. The dosage of carboplatin can be adjusted according to renal function of patients, which provides a safer therapeutic regimen for cervical cancer patients complicated with renal insufficiency. Taisuke Mori et al. evaluated the long-term efficacy of neoadjuvant chemotherapy with carboplatin plus paclitaxel combined with radical hysterectomy in the treatment of locally advanced cervical cancer (Mori et al., 2010). The therapeutic regimen had high remission rate and good postoperative tolerance, and the vast majority of adverse reactions were mild or moderate. This study provides a promising novel therapeutic approach for patients with locally advanced cervical cancer who are ineligible for primary surgery or radical radiotherapy.

Nedaplatin is another second-generation drug designed to reduce toxicity. Yang et al. evaluated the role of nedaplatin in concurrent chemoradiotherapy for cervical cancer (Yang et al., 2022). The overall survival rate and progression-free survival rate in the nedaplatin group were comparable to those in the cisplatin group, with a trend toward prolonged progression-free survival. The patients in the nedaplatin group experienced significantly reduced gastrointestinal reactions and no cases of renal failure occurred. Liu et al. first evaluated the efficacy and toxicity of nedaplatin combined with extended-field intensity-modulated radiotherapy in high-risk cervical cancer patients with para-aortic lymph node metastasis (Liu et al., 2016). Gastrointestinal reactions and renal toxicity were reduced in all patients, thus confirming the safety and efficacy of this combination strategy for such refractory patients.

### 4.3 Application of oxaliplatin and lobaplatin

The clinical value of third-generation platinum drugs lies in that they provide new hope for patients with refractory cervical cancer who are resistant or intolerant to cisplatin.

Kuo et al. investigated the efficacy and safety of paclitaxel combined with oxaliplatin in the treatment of recurrent or metastatic cervical cancer (Kuo et al., 2010). Notably, 75% of the patients had previously received cisplatin-based concurrent chemoradiotherapy, and the regimen still exhibited definite antitumor activity after treatment, with an overall response rate of 22% and a clinical benefit rate of 47%. Furthermore, patients exhibited good tolerability to the regimen; neurotoxicity was manageable via dose adjustment, and no treatment-related deaths occurred, demonstrating that the paclitaxel combined with oxaliplatin regimen holds potential application value in overcoming cisplatin resistance. Li et al. were the first to systematically investigated the antitumor activity of lobaplatin in the CaSki human cervical cancer cell line and confirmed that it could inhibit cell proliferation, induce cell cycle arrest and apoptosis in a dose- and time-dependent manner (Li et al., 2014). Tian et al. conducted a retrospective comparison of the efficacy between the lobaplatin-based (TP) regimen and cisplatin-based (TL) regimen following neoadjuvant chemotherapy (Tian et al., 2021). The survival outcomes of the TL regimen and TP regimen showed no significant difference, which confirmed their interchangeability. The adverse reactions analysis indicated that the lobaplatin regimen was associated with more pronounced hematological toxicity (specifically thrombocytopenia) but lower gastrointestinal toxicity, thereby providing important guidance for clinicians to select chemotherapy regimens for patients with different tolerance levels and those with cisplatin resistance.

## 5 Strategies to enhance therapeutic effect of platinum-based drugs

Although cisplatin and other platinum-based drugs have been widely used in the treatment of multiple cancers for many years, their therapeutic efficacy is limited, and the severe adverse effects associated with treatment have greatly restricted their clinical application. Furthermore, the clinical translation of novel platinum analogs has slowed significantly, as many candidates fail to transcend the therapeutic window constraints or overcome cross-resistance within traditional chemical frameworks. Furthermore, no new platinum-based drugs have been approved for clinical use since the third generation due to the diversity of the structure. At present, platinum-based drugs can be conjugated with inorganic materials (e.g., carbon materials, mesoporous silica nanoparticles and gold nanomaterials) and organic materials (e.g., polymers, dendrimers, and liposomal micelles) via physical encapsulation, chemical covalent bonding, or electrostatic interaction to achieve drug delivery (Kapadne et al., 2024; Kalayil et al., 2024; Stępień et al., 2025; Santos et al., 2024)Therefore, it is great significance to explore new strategies for enhancing the efficacy of platinum-based drugs. The representative platinum drugs, diverse nanocarriers, and multifunctional strategies for boosting therapeutic outcomes are summarized in Figure 3.

**FIGURE 3:**
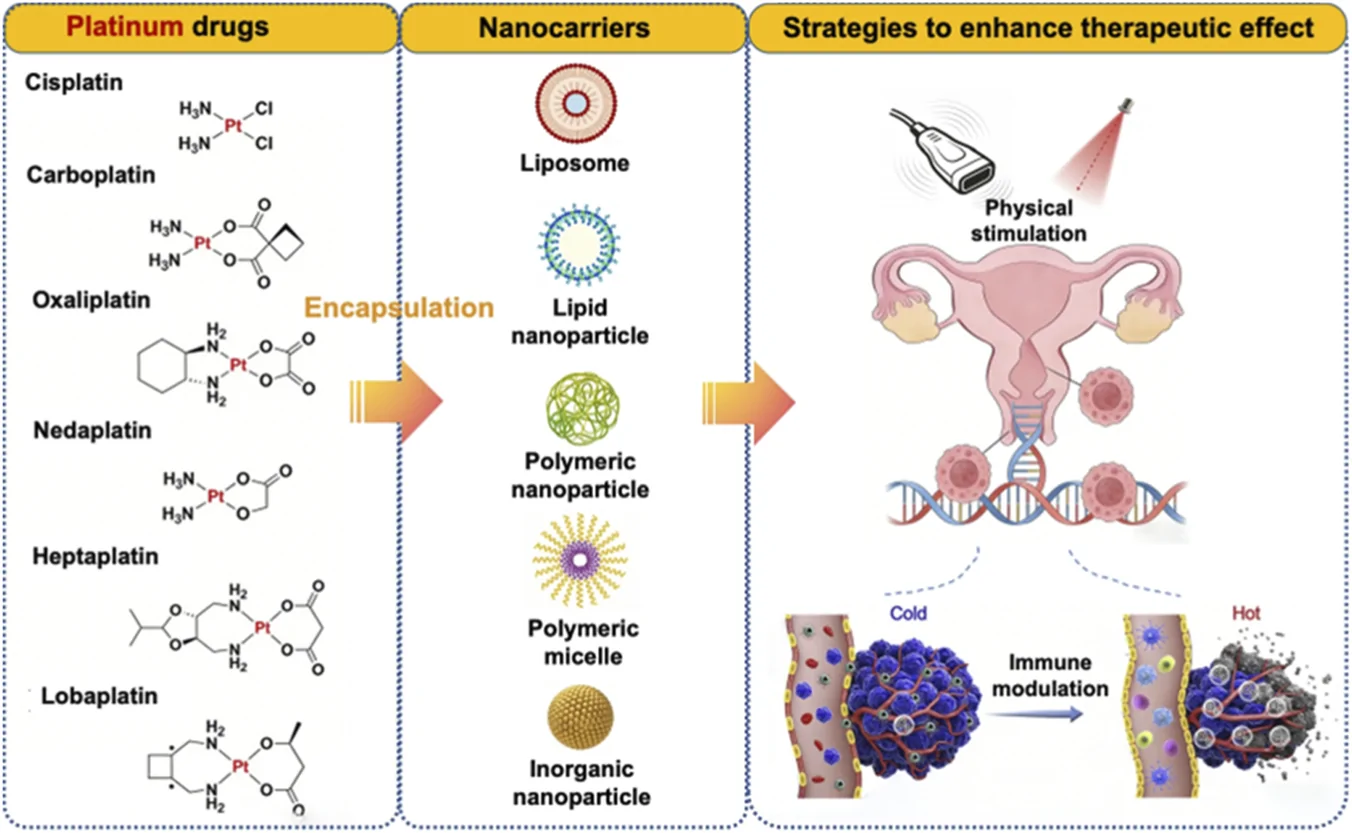
Advanced nanoplatforms and multifaceted strategies for enhancing the therapeutic effects of platinum-based chemotherapy.

### 5.1 Single platinum drug delivery system

Nanoparticles, as drug carriers, can enhance the intracellular drug concentration through effective cellular uptake and rapid drug release, thereby reversing the chemotherapy resistance of tumors (Li et al., 2023; Gao et al., 2021). Guan et al. developed a cisplatin-coupled hollow gold nanosphere for targeted therapy of cervical cancer (Guan et al., 2019). The experimental results showed that the nanocarrier could significantly enhance drug release under acidic conditions simulating the tumor environment, thereby enhancing the cytotoxicity and tumor inhibition effect of cisplatin. Luo et al. developed a nanosensitizer self-assembled from a disulfide bond-containing carrier and a platinum (IV) prodrug modified with vitamin E succinate (Luo et al., 2020). This system effectively depletes intracellular GSH, which not only reverses platinum resistance but also sensitizes cells to ROS-mediated oxidative stress, leading to mitochondrial dysfunction. This system can trigger drug release in response to the high glutathione (GSH) levels in tumor cells via glutathione-mediated activation, and effectively deplete intracellular GSH through disulfide bond exchange, thereby reversing one of the key mechanisms underlying cisplatin resistance. The nanodrug significantly enhanced platinum accumulation in drug-resistant cells and exhibited specific enrichment in mitochondria, thereby inducing mitochondrial damage, reducing ATP levels, and elevating reactive oxygen species (ROS) levels, which ultimately triggered a robust apoptotic cascade. In cisplatin-resistant cervical cancer models, the nanosystem exhibited excellent tumor-targeting ability and tumor growth inhibition efficacy, while significantly reducing the systemic toxicity of cisplatin, thus providing an efficient and safe innovative strategy for concurrent chemoradiotherapy. Wei et al. designed a novel nucleus-targeted oxaliplatin (IV) prodrug self-assembled from a molecule containing a long-chain hydrophobic lipid moiety at one end and a hydrophilic nuclear-targeting peptide (R8K) at the other (Wei et al., 2024). This system achieved a nuclear accumulation rate of platinum drugs in cancer cells as high as 74% (3.6-fold that of free oxaliplatin), and significantly promoted the formation of anticancer platinum-DNA adducts, thereby enhancing the cytotoxicity against drug-resistant cancer cells by more than 10-fold. In animal experimental models, these nanoparticles were able to effectively accumulate in tumor tissues and significantly inhibit tumor growth. Kong et al. designed a novel nano-system co-conjugating a platinum (IV) prodrug and the sonosensitizer riboflavin onto a polyglutamic acid nanocarrier (Kong et al., 2025). This system utilized ultrasound to excite riboflavin, catalyzing the generation of superoxide anions that specifically reduced platinum (IV) to the active platinum (II) form within tumor tissue. This strategy significantly increased intratumoral drug concentration and antitumor activity even under low-intensity ultrasound conditions.

### 5.2 Nano-delivery system based on combined strategy

The complex and unclear multi-drug resistance (MDR) mechanism is the main obstacle to the success of clinical cancer chemotherapy. At present, the construction of composite nanodrug delivery systems via the co-delivery of multiple chemotherapeutic agents with distinct mechanisms of action provides a novel strategy for addressing clinical multidrug resistance. Meng et al. developed a dual-locked platinum (IV) prodrug incorporating a γ-glutamyl transpeptidase-responsive Toll-like receptor 7/8 agonist (IMDQ), thereby constructing a “chemo-immunity” cascade activation platform (Meng et al., 2025). The highly expressed GGT in the tumor microenvironment cleaves and releases IMDQ, simultaneously triggering dendritic cell maturation and macrophage M1-polarization. Yang et al. constructed a platinum (IV) prodrug system integrating the DNA methyltransferase inhibitor RG108 and the histone deacetylase inhibitor phenylbutyrate (Yang et al., 2025). This system formed an epigenetic regulatory network: RG108 restores GSDME gene expression through demethylation, while phenylbutyrate enhanced the formation of platinum-DNA adducts via chromatin remodeling. The synergistic action activated the Caspase-3/GSDME-mediated pyroptosis pathway, achieving potent antimetastatic effects and reduced systemic toxicity. The transition from apoptosis to GSDME-mediated pyroptosis not only directly kills tumor cells but also triggers the release of damage-associated molecular patterns (DAMPs), thereby transforming the ‘cold’ tumor microenvironment into an ‘immunologically hot’ one. By integrating chemotherapy with epigenetic or immune regulation, these composite nanosystems address the multi-dimensional nature of tumor resistance, providing a robust framework for personalized cervical cancer therapy.

## 6 Conclusion

As a fundamental drug for anti-tumor treatment, platinum-based drugs hold an irreplaceable core position in the cervical cancer treatment system, and play a crucial role in postoperative adjuvant therapy, intervention for locally advanced lesions, and systematic management of recurrent or metastatic lesions. The traditional platinum-based drugs (e.g., cisplatin, carboplatin and oxaliplatin) have established a solid foundation of clinical efficacy. However, their inherent systemic toxicity and acquired drug resistance remain issues limit the maximization of their efficacy and their long-term application. Based on the cross-innovation of molecular pharmacology and nanotechnology, the application research of platinum-based drugs in the cervical cancer treatment has achieved multi-dimensional breakthroughs, bringing exciting opportunities for the better clinical application of platinum drugs: (i) The use of tree-like macromolecules, liposomes and metal-organic frameworks as carriers enables the precise targeted delivery and controlled release of platinum drugs, reducing systemic toxicity. (ii) The tumor-targeted delivery systems are constructed to introduce cervical cancer-specific targeting moieties (e.g., integrin-recognition peptides and folate receptor ligands), significantly enhancing the enrichment efficiency of drugs in cervical tumor tissues. At the same time, tumor microenvironment-responsive elements are integrated to enable the precise activation and release of drugs locally in tumors, thereby effectively reducing damage to normal tissues. (iii) Develop platinum-DNA cross-linking repair inhibitors, polynuclear platinum complexes, and combine them with PARP inhibitors or epigenetic drugs to break through the resistance barrier. (IV) The construction of platinum-containing radioactive probes or fluorescent markers enables precise identification of cervical tumor lesions, providing precise imaging evidence for the early diagnosis and staging assessment of cervical cancer. At the same time, it enables real-time monitoring of the dynamic changes of tumor tissues during the treatment process, achieving true integration of diagnosis and treatment. Through sustained pharmacological exploration and interdisciplinary technological integration, the future development of platinum-based drugs in the treatment of cervical cancer will continue to advance towards the directions of high efficiency, low toxicity, precision and intelligence, thereby providing better solutions to improve patient prognosis.
